# Surface charge controlled nucleoli selective staining with nanoscale carbon dots

**DOI:** 10.1371/journal.pone.0216230

**Published:** 2019-05-31

**Authors:** Zhijun Zhu, Qingxuan Li, Ping Li, Xiaojie Xun, Liyuan Zheng, Dandan Ning, Ming Su

**Affiliations:** 1 Department of Chemical Engineering, Northeastern University, Boston, Massachusetts, United States of America; 2 Wenzhou Institute of Biomaterials and Engineering, Wenzhou Medical University, and Chinese Academy of Science, Zhejiang, P. R. China; 3 School of Chemistry and Materials, Ningde Normal University, Ningde, Fujian, P. R. China; University of Puerto Rico, Rio Piedras Campus, PUERTO RICO

## Abstract

Organelle selective imaging can reveal structural and functional characters of cells undergoing external stimuli, and is considered critical in revealing biological fundamentals, designing targeted delivery system, and screening potential drugs and therapeutics. This paper describes the nucleoli targeting ability of nanoscale carbon dots (including nanodiamond) that are hydrothermally made with controlled surface charges. The surface charges of carbon dots are controlled in the range of -17.9 to -2.84 mV by changing the molar ratio of two precursors, citric acid (CA) and ethylenediamine (EDA). All carbon dots samples show strong fluorescence under wide excitation wavelength, and samples with both negative and positve charges show strong fluorescent contrast from stained nucleoli. The nucleoli selective imaging of live cell has been confirmed with Hoechst staining and nucleoli specific staining (SYTO RNA-select green), and is explained as surface charge heterogeneity on carbon dots. Carbon dots with both negative and positive charges have better ability to penetrate cell and nucleus membranes, and the charge heterogeneity helps carbon dots to bind preferentially to nucleoli, where the electrostatic environment is favored.

## Introduction

Carbon dots including nanodiamond discovered as by-products of nanotubes have shown great potentials in chemical, bio-sensing, nano-medicines, catalysis, and as active components in optical and optoelectronic devices [1–12]. The unique properties of nanoscale carbon dots such as small sizes, low toxicity, photo-stability, and rich surface chemistry make them ideal for molecular imaging [13–23]. Membrane and cytoplasm imaging has been achieved with carbon dots made from a variety of carbon sources [24–29]. Organelle selective imaging can reveal structural and functional characters of cells undergoing external stimuli, and is considered critical in revealing biological fundamentals, designing targeted delivery system, and screening potential drugs and therapeutics[30–33]. The area selective imaging of cellular organelles has been achieved in carbon dots [28]. Carbon dots made from citric acid and penicillamine can target Golgi body [34] and those from β-alanine and zwitterionic ligand can target nucleuses [35]. Developing more targeted imaging probes would definitely enhance understanding to targeted delivery, and enrich bioimaging toolkits.

Nucleoli is known for its critical roles in creation of ribosomes via ribosome biogenesis process, in assembly of signal recognition particles, and in cell’s responses to stress [36]. Though desired, nucleoli selective staining is rare, and existing methods are limited by high cost, and laborious operations[37,38]. Given their nanometer sizes, carbon dots can easily penetrate cell membranes and nucleus pores, and access to sub-cellular organizations. The rich surface chemistry of carbon dots enables unlimited possibilities of selective staining [39–43]. Carbon dots made from calcine of cow manure and modified with ethylenediamine (EDA) and from refluxing polyethylene glycol showed a tendency to stain nucleoli [44]. However, these results do not reveal the physical state of carbon dots around nucleoli, and a direct side-by-side comparison with a known nucleoli staining is needed to confirm nucleoli targeting ability.

Carbon dots made from citric acid (CA) and ethylenediamine (EDA) have also been studied for bioimaging [45,46]. However, the influence of surface charge to nucleoli staining ability has not been reported. Here, CA and EDA, are used simultaneously to produce carbon dots with controlled surface charge under hydrothermal conditions. The surface charges of carbon dots have been tuned by adjusting the molar ratio of CA and EDA to achieve impressive nucleoli targeting with high selectivity. While decreasing the molar ratio of CA: EDA in precursor, the zeta potential of carbon dots decreases accordingly, which enhances nucleoli staining. The effects of incubation time and carbon dot concentration have been examined. The nucleoli selective staining of these carbon dots has been observed in different cell line, and thus can be extended to other cell lines in general.

## Experimental sections

Chemicals: Citric acid (CA), and polyethylenimine (PEI) were purchased form Alfa Aesar. Ethylenediamine (EDA), Dulbecco’s modified Eagle’s medium (DMEM, Hyclone), fetal bovine serum (FBS), penicillin and streptomycin were obtained from Corning. SYTO RNA-select green fluorescent cell stain and Hoechst 33342 were from ThermoFisher. Phosphate buffer solution (PBS) was obtained from ThermoFisher. Others were from Sigma-Aldrich. All the chemicals were used as received. Milli-Q water (Millipore) was used to make all the solutions.

Carbon dots preparation: carbon dots were made according to literature [45] with slight modification. Briefly, 1.6 g of CA (1 mol) was dissolved in 10 mL of water, and mixed with 0.21 (0.5 mol), 0.42 (1 mol), 0.83 (2 mol) or 1.66 mL (3 mol) of EDA, respectively, followed by stirring to form a homogeneous solution. The mixture was transferred to a 50 mL of Teflon-lined stainless autoclave and heated at 180°C for 12 h. After reaction, the reactor was cooled down to room temperature naturally. The resulting solution was subjected to dialysis with molecular weight cut-off (MWCO) of 1000 to remove unreacted precursors, followed by lyophilization. Two carbon dots had also been synthesized using CA as carbon resource and PEI as a passivation agent, where the molar ratio of CA: PEI was 1:0.5 and 1:2, respectively. The carbon dots made using CA and EDA as a single carbon resource were used as controls. Measurement: The fluorescence spectra were measured with Cary Eclipse Fluorescence Spectrophotometer. Fourier-transform infrared (FTIR) spectroscopy was conducted on a Shimadzu IR Prestige21 spectrometer. The morphology was observed on a JEM-1010 Transmission Electron Microscope (TEM) operating at 60 kV using ultrathin carbon supported grid. A Renishaw Raman spectrometer (Invia) was operated at 532 nm excitation wavelength to obtain Raman spectrum of each sample. Zeta potential was measured on a NANO ZS ZEN3600, Malvern Instruments. The photoluminescence (PL) quantum yield (QY) of the carbon dots with the different molar ratio of CA:EDA were derived with the following Eq (1) [47]:

$${QY}_{CDs}={QY}_{R}\frac{I_{CDs}A_{R}{\left(n_{CDs}\right)}^{2}}{I_{R}A_{CDs}{\left(n_{R}\right)}^{2}}$$ (1)

where, QY_*CDs*_ and QY_*R*_ are the PL QY of the CDs and reference (Rhodamine 6G), the QY_R_ of Rhodamine 6G in water solution is 0.95[48]. I_*CDs*_ and I_*R*_ is the measured integrated emission intensity of CDs and reference, respectively. A_*CDs*_ and A_*R*_ is the UV-vis absorption intensity of CDs and reference, respectively. n_*CDs*_ and n_*R*_ is the refractive index of the solvent (1.33 for water) for both CDs and reference.

Cell culture and viability test: HeLa (human cervical cancer) cells were cultured in 5% CO_2_ at 37°C in DMEM supplemented with 10% FBS and 1% mixed solution of penicillin and streptomycin. For cell viability test, 8,000 HeLa cells were seeded in 96 well plates overnight. Desired amounts of carbon dots were added to the media, and the cells were incubated with another 24 h. The cells were washed thrice with PBS (pH 7.4), and then the viability of the cells were tested with MTT method.

Cell staining and imaging: 100,000 cells were seeded in 12-well plate and incubated overnight with carbon dots at certain concentration. After certain time, the cells were washed with PBS twice and fixed with 4% formaldehyde for 15 min in an incubator. 1 mL of DMEM that contains 16.2 μM of Hoechst 33342 and 0.5 μM of RNA-select green fluorescent cell stain was applied on the cells and incubated for another 20 min in dark. After removing the solution, the cells were rinsed twice with fresh DMEM, and the cells were rest for 5 minutes in medium at 37°C. The coverslip was taken out and inversely placed on a glass slide pre-dropped with mounting medium (a mixture of 30% 2×PBS and 70% glycerin) and the cells were ready for imaging.

## Results and discussions

**Fig 1:**
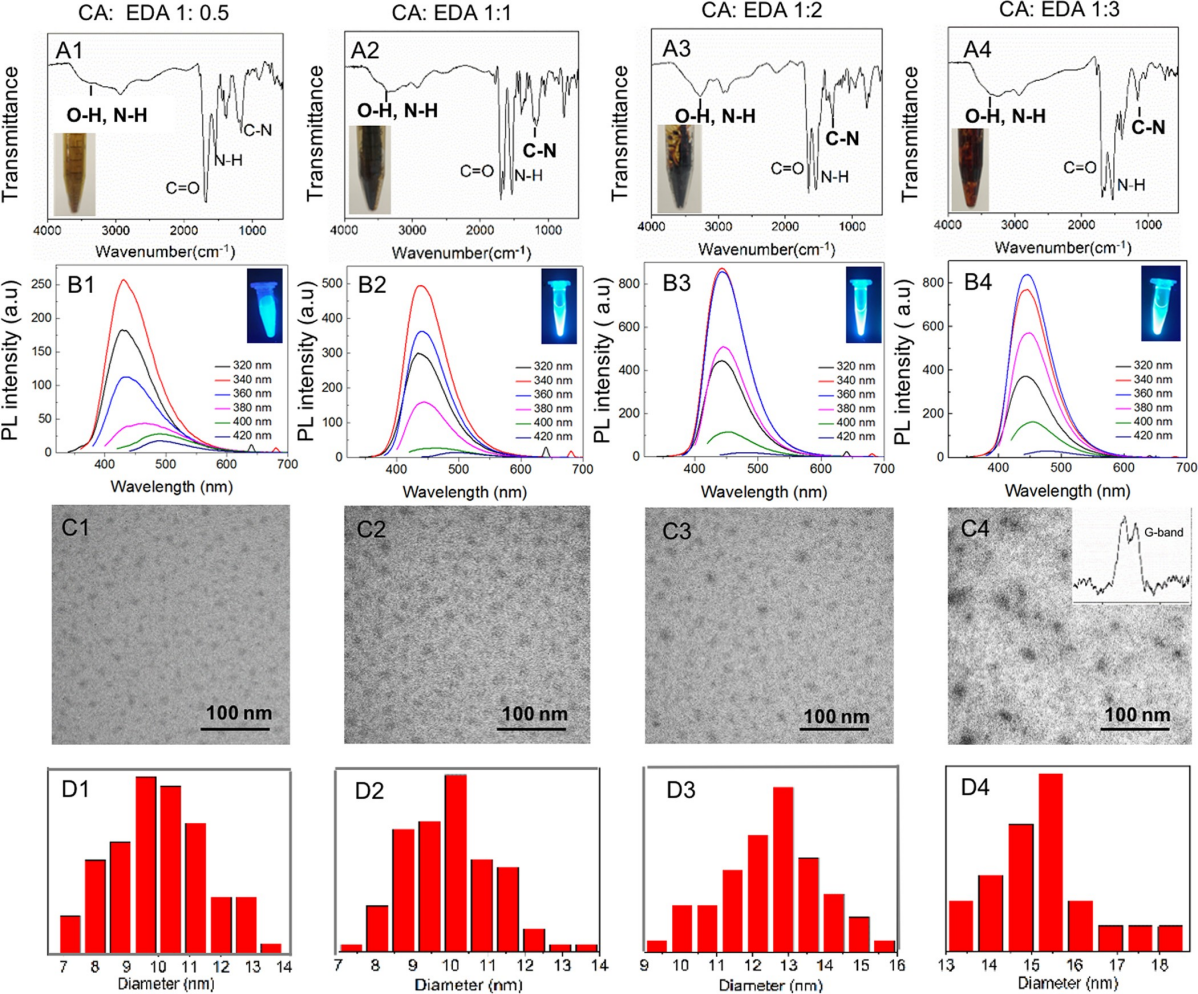
FTIR spectrum (A), fluorescent emission spectra at the same concentration (B), and TEM (C) of carbon dots made from different molar ratio of CA: EDA. The Raman spectrum of one sample is also included as inset in C4.

The hydrothermal method has been used to make carbon dots from various carbon sources Fourier transform infrared spectroscopy (FTIR) provides abundant information of the surface groups of carbon dots (Fig 1A1–1A4), where the broad peaks centered at 3440 cm^-1^ correspond to the stretching vibration of O-H and N-H bonds, respectively. Both O-H and N-H bonds improve the hydrophilicity and stability of carbon dots in an aqueous solution. The strong peaks at 1700 and 1200 cm^-1^ are attributed to the stretching vibration of C = O and C-N bonds, while that at 1600 cm^-1^ to the bending vibration of N-H bond. With the decrease of CA: EDA, the signal intensity of N-H bond increases relative to that of C = O bond, which indication that relatively more EDAs are involved. The FTIR results also indicate that these as-made carbon dots possess abundant hydroxyl and carboxylic groups, which ensure their good biocompatibility.

The surface charges around carbon dots enable excellent dispersion in aqueous solution. Fig 1B1–1B4 inset shows the aqueous solutions of four carbon dots emit bright blue light under ultraviolet excitation (365 nm) and also the PL spectra of the four aqueous solutions of carbon dots with excitation wavelength from 320 to 420 nm. The emission spectra of carbon dots synthesized from CA and EDA exhibit excitation-wavelength dependence, and the narrow emission peak reflects narrow size distribution of carbon dots. At the same CA: EDA molar ratio, the emission peak shifts to long wavelength (red shift) at long excitation wavelength, and the peak intensity decreases sharply when excitation wavelength is above 400 nm. Unlike a simple organic fluorophore that has one excitation level in visible region, carbon dots have multiple excitation levels in the visible region, and show excitation dependent emission[49].

Fig 1C shows transmission electron microscopy (TEM) images of carbon dots deposited on ultrathin carbon films supported on copper grid. The carbon dots made from different molar ratio of CA: EDA show a uniform size distribution (Fig 1C1–1C4) with diameter of carbon dots increasing from 10 to 15 nm. The samples have been tested for their Raman characteristics under 532 nm excitation. The fourth sample with less amount of negative charges shows strong sp^3^ diamond peak at 1332 cm^-1^, while the other three samples with more negative charges do not show the peak (Figure not shown). This formation of crystalline diamond structure could also be indicated from the higher electron contrast in TEM image (Fig 1C4) than other three samples. The PL QY of carbon dots made from different molar ratio of CA: EDA were calculated, the QY of CA: EDA(1:0.5), CA: EDA(1:1), CA: EDA(1:2) and CA: EDA(1:3) was around 0.87, 0.84, 0.89 and 0.84, respectively. These four carbon dots exhibit high PL QY.

**Fig 2:**
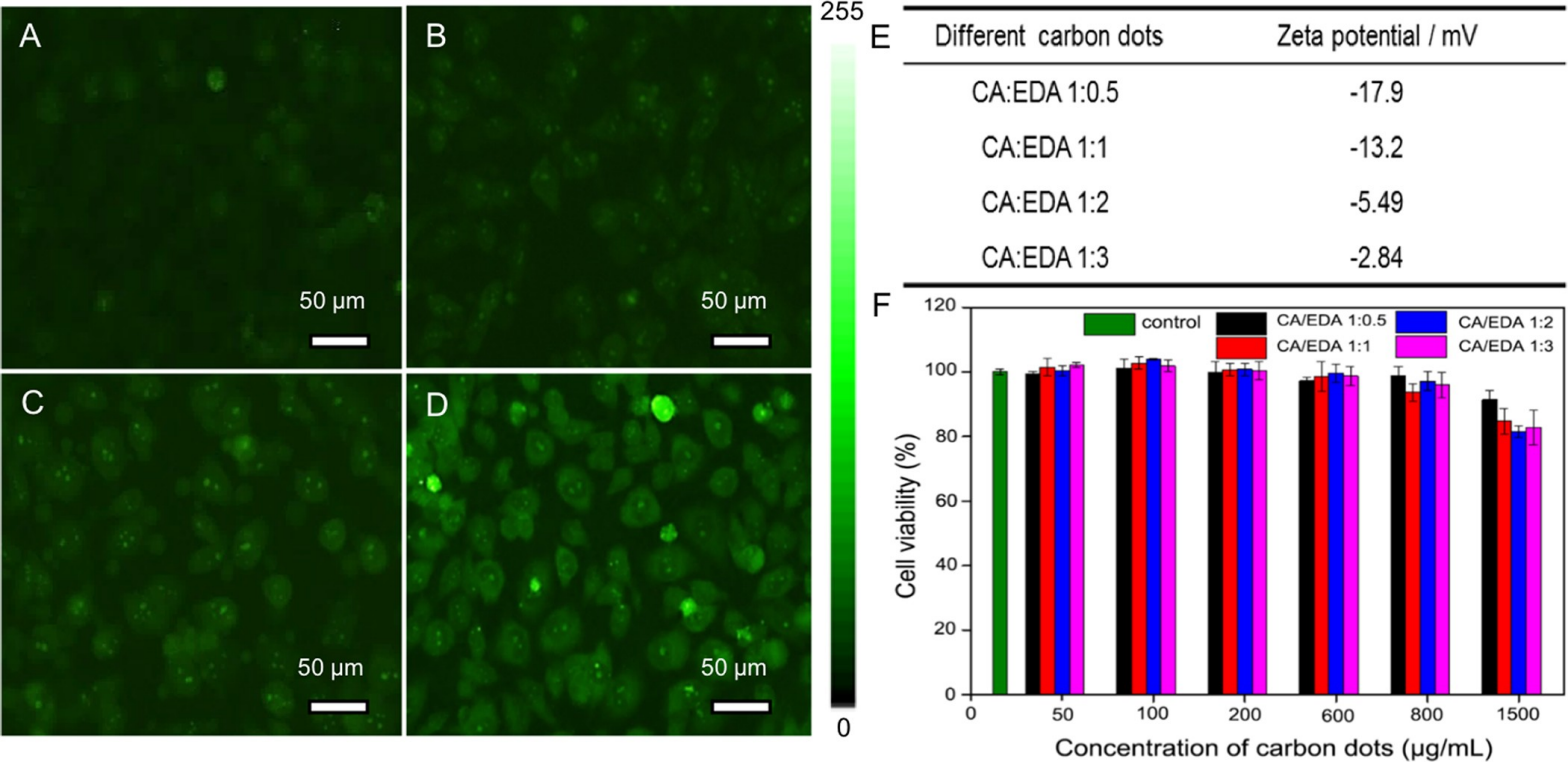
Fluorescent images of HeLa cells incubated with 400 μg/mL of carbon dots with the molar ratio of CA: EDA at 1:0.5(A), 1:1 (B), 1:2 (C), 1:3 (D) for 24 h (under blue light excitation). Table of the Zeta potentials of carbon dots with different molar ratio of CA: EDA (E). Viability of HeLa cells incubated with various carbon dots with different concentrations for 24 h (F).

Fig 2A–2D shows cell outlines observed using carbon dots of different CA: EDA, where a trend of strong image contrast can be seen when the CA: EDA ratio increases from 1:0.5 (Fig 2A), 1:1 (Fig 2B), 1:2 (Fig 2C) and 1:3 (Fig 2D), respectively. Since fluorescent intensity of carbon dots from different molar ratios show the same according to PL spectra in Fig 1B, the best local selective staining ability, which can be seen from Fig 2C and 2D, indicates more carbon dots aggregated in the centre of nucleus. The selective staining ability is quantified by image according to intensity differences between nucleoli and cytoplasm, which shows higher intensity difference in Fig 2C (24) and 2D (18) than that in Fig 2A (0) and 2B (8), proving the possible ability of selectivity. The following nucleoli staining test was practiced at CA: EDA molar ratio of 1:2 rather than 1:3 to achieve sufficient contrast while avoiding heavily staining which may alter cellular functions. The Table in Fig 2E shows the Zeta potentials of carbon dots with different molar ratio of CA: EDA, where the zeta potential increased with the molar ratio of CA: EDA decreased, meaning more amine groups are presented on the surface of carbon dots. Figures of Zeta potential are presented in S1 Fig. Compared Zeta potential and staining ability among these four samples, the increasing trend of Zeta potential is correlated to the ability of staining nucleoli due to cell uptake affinity of positive or neutral species[50]. The in vitro cellular toxicity of the carbon dots was examined with MTT method. Fig 2F shows that cells exposed to four different carbon dots showed good viability over a large concentration range after 24 h incubation, where no evident toxicity was observed at 800 μg/mL of carbon dots, and over 80% of cells remain alive at 1,500 μg/mL of carbon dots, indicating excellent biocompatibility of carbon dots. The concentration of carbon dots in the staining solution was thus determined to be 400 μg/mL.

**Fig 3:**
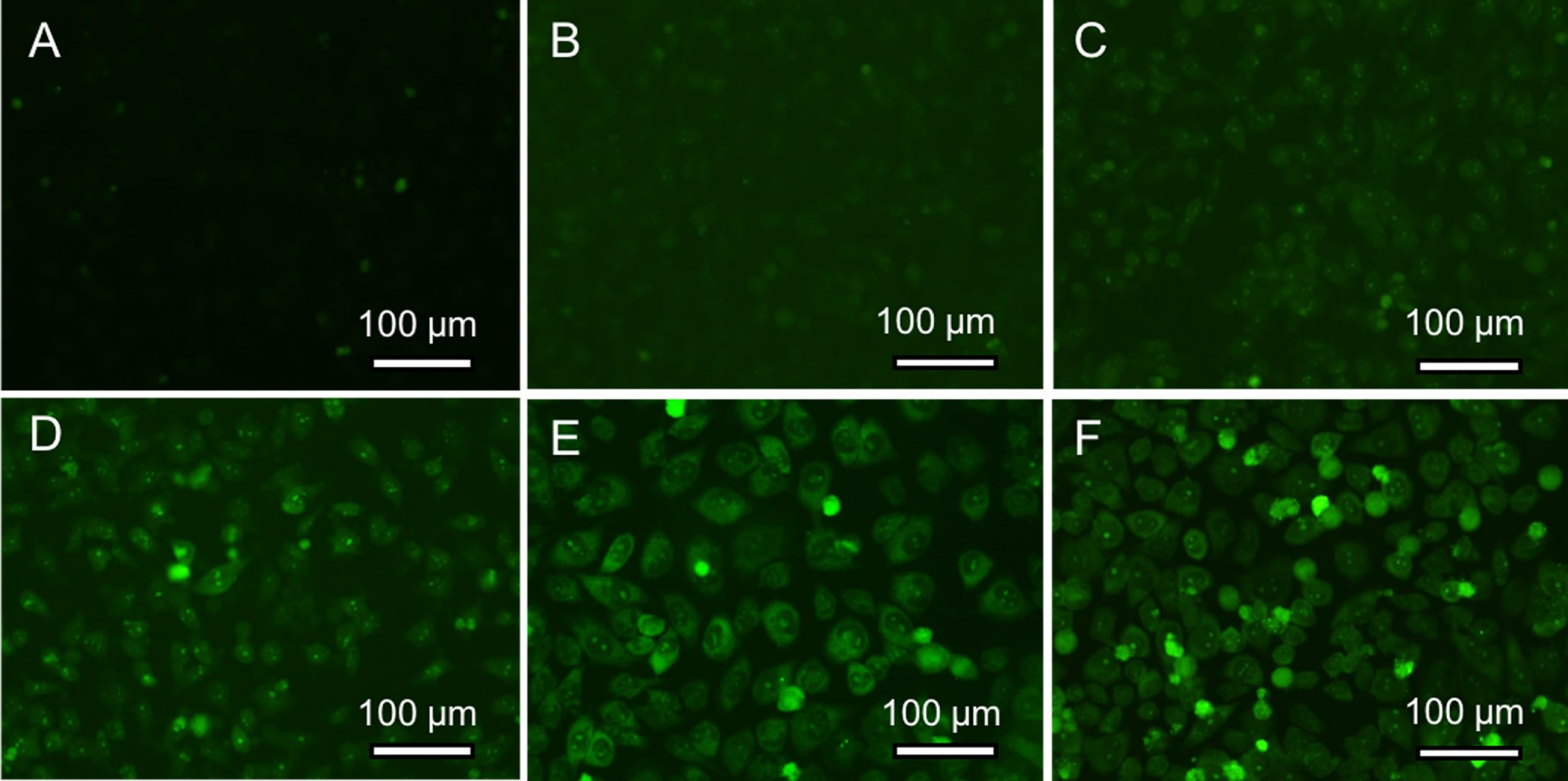
The fluorescent images of HeLa cells incubated with 400 μg/mL of carbon dots from CA: EDA 1:2 for 0 h (A), 1 h (B), 2 h (C), 4 h (D), 8 h (E) and 24 h (F), respectively (under blue excitation).

Cell incubated with 400 μg/mL of carbon dots at CA: EDA ratio of 1:2 for different time was investigated to determine the suitable incubation time for selective staining ability. Fig 3 shows the gradual enrichment of carbon dots around nucleoli during a 24 h period of time, where the nucleoli become brighter and clearer as the incubation time increases. No obvious nucleoli image can be recognized before (Fig 3A) and after incubation for 1 h (Fig 3B); vague outline of nucleoli with weak contrast can be seen after staining for 2 h (Fig 3C); clear nucleoli with strong contrast can be seen after staining for 4 h and 8 h (Fig 3D and 3E). Since exposure to 400 μg/mL of carbon dots for 24 h does not lead to significant higher contrast of nucleoli and nucleus (Fig 3F), staining for 4 h is determined to be appropriate for the further experiments.

**Fig 4:**
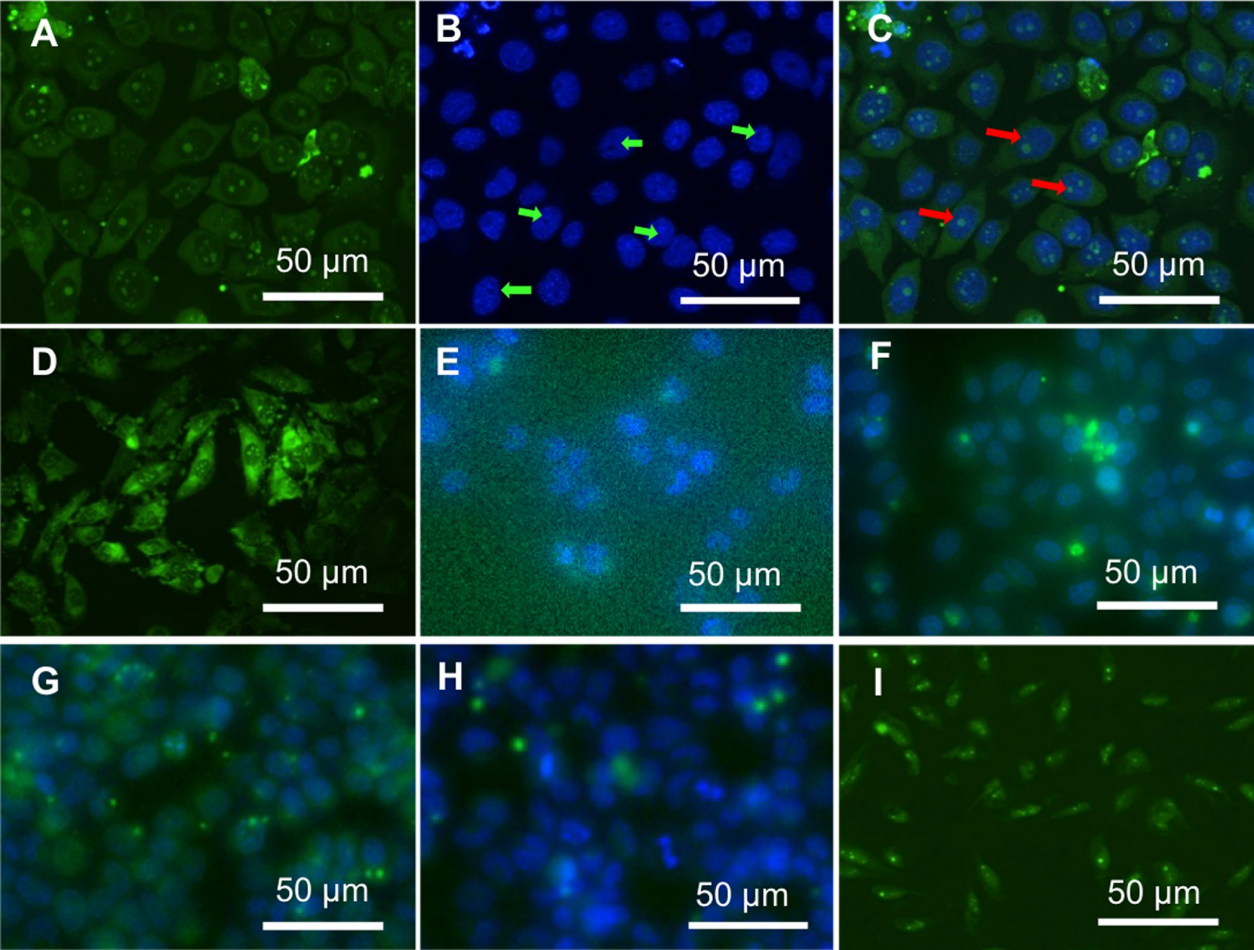
The fluorescent image of HeLa cells incubated with carbon dots from CA: EDA 1:2 and Hoechst 33342, (A) for blue bright excitation, (B) for UV bright excitation, (C) for overlap of (A) and (B). Fluorescent image of the HeLa cells stained with SYTO RNA-select green fluorescent dye (D). The fluorescent images of HeLa cell incubated with 400 μg/mL of carbon dots made from CA (E), EDA (F), and CA and PEI with CA: EDA 1:0.5 (G) and 1:2 (H) for 24 h. The fluorescent images of LN229 incubated with 400 μg/mL of carbon dots for 4 h (I).

In order to specific determine the location of carbon dots inside cells, cells are co-stained with carbon dots (CA: EDA molar ratio of 1:2), and nucleus selective dye that tends to bind A-T rich regions of DNA (Hoechst 33342), and commercial RNA selective dye (SYTO RNA-select green fluorescent cell stain) that has been known the capability of RNA staining is provided as a comparison. Fig 4A–4C shows fluorescent images of cells co-stained with carbon dots from CA: EDA 1:2 and Hoechst 33342.

Fig 4A shows that cells emit bright green fluorescence upon blue excitation due to presence of carbon dots. Fig 4B shows the same cells emit blue fluorescence of Hoechst 33342, which shows void spots in nucleus as nucleoli (indicated by green arrows) because there is not enough DNAs in nucleoli region. The bright green spots in the nucleus match void regions perfectly, suggesting that carbon dots are selective staining agents for live cell nucleoli. Since nucleolus is composed of proteins, ribosomal RNA (rRNA) and rDNA, cells were stained with commercial RNA selective dye (SYTO RNA-select green fluorescent cell stain) to further confirm that carbon dots have high selectivity to nucleoli. The fact shown in Fig 4D that the fluorescence image of cells stained with SYTO dye is virtually non-distinguishable from those of carbon dots, confirming that the carbon dots have a strong affinity to cell nucleoli. The above nucleoli staining with carbon dots suggests that carbon dots with less negative charge (lower CA: EDA ratio) provide better selectivity. To clarify the cause, CA and EDA were used to prepare carbon dots respectively as controls. Figure of PL spectra in S2 Fig. indicate that carbon dots from CA shows much lower intensity than those from EDA. Cells had been treated with the two carbon dots, respectively. Carbon dots from CA or EDA does not show selective staining ability (Fig 4E and 4F, respectively), indicating that the nucleoli selectivity is achieved only when both EDA and CA are used. In order to test whether a combination of PEI and CA would contribute to the selectivity, carbon dots at two different CA: PEI ratios were used to stain cells. Both carbon dots of different CA: PEI show high PL intensity and can enter cells (Fig 4G and 4H), but without any selectivity. This fact proves that the nucleoli staining ability is strongly related to carbon dots derived from different carbon source. The selective nucleoli staining with surface charge controlled carbon dots is not limited to HeLa Cells. Fig 4I shows the fluorescence image of LN229 (glioblastoma) cells stained with carbon dots at the CA: EDA ratio of 1:3 for 4 h, where bright green emission from nucleoli of LN229 cells confirms the general applicability of carbon dots in selective nucleoli staining. The selectivity only presented in the case of both EDA and CA can be explained as surface charge heterogeneity on carbon dots. Given appropriate sizes, nanoparticles with both negative and positive surface charges may have better ability to penetrate cell and nucleus membranes. The charge heterogeneity also helps carbon dots to bind preferentially to nucleoli regions, where the electrostatic environment is favoured.

## Conclusions

Surface charge of carbon dots plays a significant role in selective nucleoli staining and imaging. Fluorescence carbon dots were made using CA as carbon source and EDA as surface modification under hydrothermal conditions. The surface charges of carbon dots are controlled in the range of -17.9 to -2.84 mV by changing the molar ratio of two precursors. All carbon dots samples show strong fluorescence and excitation-dependent photoluminescence, and samples with less negative charge show characteristic Raman peaks of nano-diamond. Remarkable selective nucleoli staining of HeLa and LN229 cell lines is found when both negative and positive charges are presented on carbon dots. The selective nucleoli staining has been confirmed by co-staining with carbon dots, Hoechst dye and RNA specific dye. In contrast, carbon dots made from citric acid and urea with more negative charge do not show selective nucleoli imaging ability. In addition, carbon dots of different surface charge have shown low toxicity and good cell compatibility.

## Supporting information

S1 Fig Zeta potentials of carbon dots with different molar ratio of CA: EDA at pH 7.0 at 25°C. (TIF)

S2 Fig The emission spectra of different carbon dots under 349 nm of excitation wavelength. (TIF)

## References

[1] Liu W, Li C, Ren Y, Sun X, Pan W, et al (2016) Carbon dots: surface engineering and applications. J Mater Chem B 4: 5772–5788. 10.1039/c6tb00976j 32263748

[2] Pierrat P, Wang R, Kereselidze D, Lux M, Didier P, et al (2015) Efficient in vitro and in vivo pulmonary delivery of nucleic acid by carbon dot-based nanocarriers. Biomaterials 51: 290–302. 10.1016/j.biomaterials.2015.02.017 25771019

[3] Lim SY, Shen W, Gao Z (2015) Carbon quantum dots and their applications. Chem Soc Rev 44: 362–381. 10.1039/c4cs00269e 25316556

[4] Kong B, Zhu A, Ding C, Zhao X, Li B, et al (2012) Carbon dot-based inorganic–organic nanosystem for two-photon imaging and biosensing of pH variation in living cells and tissues. Adv Mater 24: 5844–5848. 10.1002/adma.201202599 22933395

[5] Kim J, Park J, Kim H, Singha K, Kim WJ (2013) Transfection and intracellular trafficking properties of carbon dot-gold nanoparticle molecular assembly conjugated with PEI-pDNA. Biomaterials 34: 7168–7180. 10.1016/j.biomaterials.2013.05.072 23790437

[6] Tao H, Yang K, Ma Z, Wan J, Zhang Y, et al (2012) In vivo NIR fluorescence imaging, biodistribution, and toxicology of photoluminescent carbon dots produced from carbon nanotubes and graphite. Small 8: 281–290. 10.1002/smll.201101706 22095931

[7] Jiang K, Sun S, Zhang L, Lu Y, Wu A, et al (2015) Red, green, and blue luminescence by carbon dots: full-color emission tuning and multicolor cellular imaging. Angew Chem Int Ed 54: 5360–5363. 10.1002/anie.201501193 25832292

[8] Gupta V, Chaudhary N, Srivastava R, Sharma GD, Bhardwaj R, et al (2011) Luminscent graphene quantum dots for organic photovoltaic devices. J Am Chem Soc 133: 9960–9963. 10.1021/ja2036749 21650464

[9] Yang S-T, Cao L, Luo PG, Lu F, Wang X, et al (2009) Carbon dots for optical imaging in vivo. J Am Chem Soc 131: 11308–11309. 10.1021/ja904843x 19722643 PMC2739123

[10] Yan F, Zou Y, Wang M, Mu X, Yang N, et al (2014) Highly photoluminescent carbon dots-based fluorescent chemosensors for sensitive and selective detection of mercury ions and application of imaging in living cells. Sens Actuators B: Chem 192: 488–495.

[11] Sharma V, Tiwari P, Mobin SM (2017) Sustainable carbon-dots: recent advances in green carbon dots for sensing and bioimaging. J Mater Chem B 5: 8904–8924. 10.1039/c7tb02484c 32264117

[12] Jiang D, Chen Y, Li N, Li W, Wang Z, et al (2016) Synthesis of Luminescent Graphene Quantum Dots with High Quantum Yield and Their Toxicity Study. PLOS ONE 10: e0144906. 10.1371/journal.pone.0144906 PMC4699207 26709828

[13] Kong W, Liu R, Li H, Liu J, Huang H, et al (2014) High-bright fluorescent carbon dots and their application in selective nucleoli staining. J Mater Chem B 2: 5077–5082. 10.1039/c4tb00579a 32261841

[14] Qu K, Wang J, Ren J, Qu X (2013) Carbon dots prepared by hydrothermal treatment of dopamine as an effective fluorescent sensing platform for the label‐free detection of iron (III) ions and dopamine. Chem Eur J 19: 7243–7249. 10.1002/chem.201300042 23576265

[15] Du F, Zhang L, Zhang L, Zhang M, Gong A, et al (2017) Engineered gadolinium-doped carbon dots for magnetic resonance imaging-guided radiotherapy of tumors. Biomaterials 121: 109–120. 10.1016/j.biomaterials.2016.07.008 28086179

[16] Wang S, Li C, Qian M, Jiang H, Shi W, et al (2017) Augmented glioma-targeted theranostics using multifunctional polymer-coated carbon nanodots. Biomaterials 141: 29–39. 10.1016/j.biomaterials.2017.05.040 28666100

[17] Wu Y-F, Wu H-C, Kuan C-H, Lin C-J, Wang L-W, et al (2016) Multi-functionalized carbon dots as theranostic nanoagent for gene delivery in lung cancer therapy. Sci Rep 6: 21170 10.1038/srep21170 26880047 PMC4754752

[18] Feng T, Ai X, An G, Yang P, Zhao Y (2016) Charge-convertible carbon dots for imaging-guided drug delivery with enhanced in vivo cancer therapeutic efficiency. ACS Nano 10: 4410–4420. 10.1021/acsnano.6b00043 26997431

[19] Vázquez-González M, Liao W-C, Cazelles R, Wang S, Yu X, et al (2017) Mimicking horseradish peroxidase functions using Cu2+-modified carbon nitride nanoparticles or Cu2+-modified carbon dots as heterogeneous catalysts. ACS Nano 11: 3247–3253. 10.1021/acsnano.7b00352 28234445

[20] LeCroy GE, Sonkar SK, Yang F, Veca LM, Wang P, et al (2014) Toward structurally defined carbon dots as ultracompact fluorescent probes. ACS Nano 8: 4522–4529. 10.1021/nn406628s 24702526

[21] Hola K, Zhang Y, Wang Y, Giannelis EP, Zboril R, et al (2014) Carbon dots—Emerging light emitters for bioimaging, cancer therapy and optoelectronics. Nano Today 9: 590–603.

[22] Zeng Q, Shao D, He X, Ren Z, Ji W, et al (2016) Carbon dots as a trackable drug delivery carrier for localized cancer therapy in vivo. J Mater Chem B 4: 5119–5126. 10.1039/c6tb01259k 32263509

[23] Wang L, Li B, Xu F, Li Y, Xu Z, et al (2017) Visual in vivo degradation of injectable hydrogel by real-time and non-invasive tracking using carbon nanodots as fluorescent indicator. Biomaterials 145: 192–206. 10.1016/j.biomaterials.2017.08.039 28869865

[24] Yang Y, Gao C, Li B, Xu L, Duan L (2014) A rhodamine-based colorimetric and reversible fluorescent chemosensor for selectively detection of Cu2+ and Hg2+ ions. Sensors and Actuators B: Chemical 199: 121–126.

[25] Luo PG, Sahu S, Yang S-T, Sonkar SK, Wang J, et al (2013) Carbon "quantum" dots for optical bioimaging. J Mater Chem B 1: 2116–2127. 10.1039/c3tb00018d 32260843

[26] Cao L, Wang X, Meziani MJ, Lu F, Wang H, et al (2007) Carbon dots for multiphoton bioimaging. J Am Chem Soc 129: 11318–11319. 10.1021/ja073527l 17722926 PMC2691414

[27] Hu L, Sun Y, Li S, Wang X, Hu K, et al (2014) Multifunctional carbon dots with high quantum yield for imaging and gene delivery. Carbon 67: 508–513.

[28] Fowley C, McCaughan B, Devlin A, Yildiz I, Raymo FM, et al (2012) Highly luminescent biocompatible carbon quantum dots by encapsulation with an amphiphilic polymer. Chem Comm 48: 9361–9363. 10.1039/c2cc34962k 22892652

[29] Mewada A, Pandey S, Thakur M, Jadhav D, Sharon M (2014) Swarming carbon dots for folic acid mediated delivery of doxorubicin and biological imaging. J Mater Chem B 2: 698–705. 10.1039/c3tb21436b 32261288

[30] Yang G, Liu L, Yang Q, Lv F, Wang S (2012) A Multifunctional Cationic Pentathiophene: Synthesis, Organelle-Selective Imaging, and Anticancer Activity. Advanced Functional Materials 22: 736–743.

[31] Senthilnathan N, Chandaluri CG, Radhakrishnan TP (2017) Efficient Bioimaging with Diaminodicyanoquinodimethanes: Selective Imaging of Epidermal and Stomatal Cells and Insight into the Molecular Level Interactions. Scientific Reports 7: 10583 10.1038/s41598-017-11293-y 28878252 PMC5587692

[32] Hu F, Liu B (2016) Organelle-specific bioprobes based on fluorogens with aggregation-induced emission (AIE) characteristics. Organic & Biomolecular Chemistry 14: 9931–9944. 27779629 10.1039/c6ob01414c

[33] Zhu H, Fan J, Du J, Peng X (2016) Fluorescent Probes for Sensing and Imaging within Specific Cellular Organelles. Accounts of Chemical Research 49: 2115–2126. 10.1021/acs.accounts.6b00292 27661761

[34] Liu X, Pang J, Xu F, Zhang X (2016) Simple approach to synthesize amino-functionalized carbon dots by carbonization of chitosan. Sci Rep 6: 31100 10.1038/srep31100 27492748 PMC4974616

[35] Jung YK, Shin E, Kim B-S (2015) Cell nucleus-targeting zwitterionic carbon dots. Sci Rep 5: 18807 10.1038/srep18807 26689549 PMC4686939

[36] Shen R, Shen X, Zhang Z, Li Y, Liu S, et al (2010) Multifunctional conjugates to prepare nucleolar-targeting CdS quantum dots. J Am Chem Soc 132: 8627–8634. 10.1021/ja1002668 20518506

[37] Wang X, Wang Y, He H, Ma X, Chen Q, et al (2017) Deep-Red Fluorescent Gold Nanoclusters for Nucleoli Staining: Real-Time Monitoring of the Nucleolar Dynamics in Reverse Transformation of Malignant Cells. ACS Applied Materials & Interfaces 9: 17799–17806. 10.1021/acsami.7b04576 28492304

[38] Saini AK, Sharma V, Mathur P, Shaikh MM (2016) The development of fluorescence turn-on probe for Al(III) sensing and live cell nucleus-nucleoli staining. Scientific Reports 6: 34807 10.1038/srep34807 27721431 PMC5056391

[39] Wang F, Xie Z, Zhang H, Liu Cy, Zhang Yg (2011) Highly luminescent organosilane‐functionalized carbon dots. Adv Funct Mater 21: 1027–1031.

[40] Salinas-Castillo A, Ariza-Avidad M, Pritz C, Camprubi-Robles M, Fernandez B, et al (2013) Carbon dots for copper detection with down and upconversion fluorescent properties as excitation sources. Chem Comm 49: 1103–1105. 10.1039/c2cc36450f 23283251

[41] Bhunia SK, Maity AR, Nandi S, Stepensky D, Jelinek R (2016) Imaging cancer cells expressing the folate receptor with carbon dots produced from folic acid. ChemBioChem 17: 614–619. 10.1002/cbic.201500694 26773979

[42] Wang C-I, Periasamy AP, Chang H-T (2013) Photoluminescent C-dots@RGO probe for sensitive and selective detection of acetylcholine. Anal Chem 85: 3263–3270. 10.1021/ac303613d 23398232

[43] Huang P, Lin J, Wang X, Wang Z, Zhang C, et al (2012) Light‐triggered theranostics based on photosensitizer‐conjugated carbon dots for simultaneous enhanced‐fluorescence imaging and photodynamic therapy. Adv Mater 24: 5104–5110. 10.1002/adma.201200650 22718562 PMC3657566

[44] D'Angelis do E S. Barbosa C, Corrêa JR, Medeiros GA, Barreto G, Magalhães KG, et al (2015) Carbon dots (C‐dots) from cow manure with impressive subcellular selectivity tuned by simple chemical modification. Chem Eur J 21: 5055–5060. 10.1002/chem.201406330 25693878

[45] He H, Wang Z, Cheng T, Liu X, Wang X, et al (2016) Visible and Near-Infrared Dual-Emission Carbogenic Small Molecular Complex with High RNA Selectivity and Renal Clearance for Nucleolus and Tumor Imaging. ACS Appl Mater Interfaces 8: 28529–28537. 10.1021/acsami.6b10737 27704754

[46] Song Y, Zhu S, Zhang S, Fu Y, Wang L, et al (2015) Investigation from chemical structure to photoluminescent mechanism: a type of carbon dots from the pyrolysis of citric acid and an amine. J Mater Chem C 3: 5976–5984.

[47] Wang L, Yin Y, Jain A, Zhou HS (2014) Aqueous Phase Synthesis of Highly Luminescent, Nitrogen-Doped Carbon Dots and Their Application as Bioimaging Agents. Langmuir 30: 14270–14275. 10.1021/la5031813 25365539

[48] Magde D, Rojas GE, Seybold PG (1999) Solvent Dependence of the Fluorescence Lifetimes of Xanthene Dyes. Photochemistry and Photobiology 70: 737–744.

[49] Zhu S, Song Y, Zhao X, Shao J, Zhang J, et al (2015) The photoluminescence mechanism in carbon dots (graphene quantum dots, carbon nanodots, and polymer dots): current state and future perspective. Nano Research 8: 355–381.

[50] Mosquera J, García I, Liz-Marzán LM (2018) Cellular Uptake of Nanoparticles versus Small Molecules: A Matter of Size. Accounts of Chemical Research 51: 2305–2313. 10.1021/acs.accounts.8b00292 30156826

