# Supplementary figures and images for: Surface charge controlled nucleoli selective staining with nanoscale carbon dots
Source: PLoS One. 2019 May 31;14(5):e0216230. doi: 10.1371/journal.pone.0216230 (PMC6544201; doi:10.1371/journal.pone.0216230)

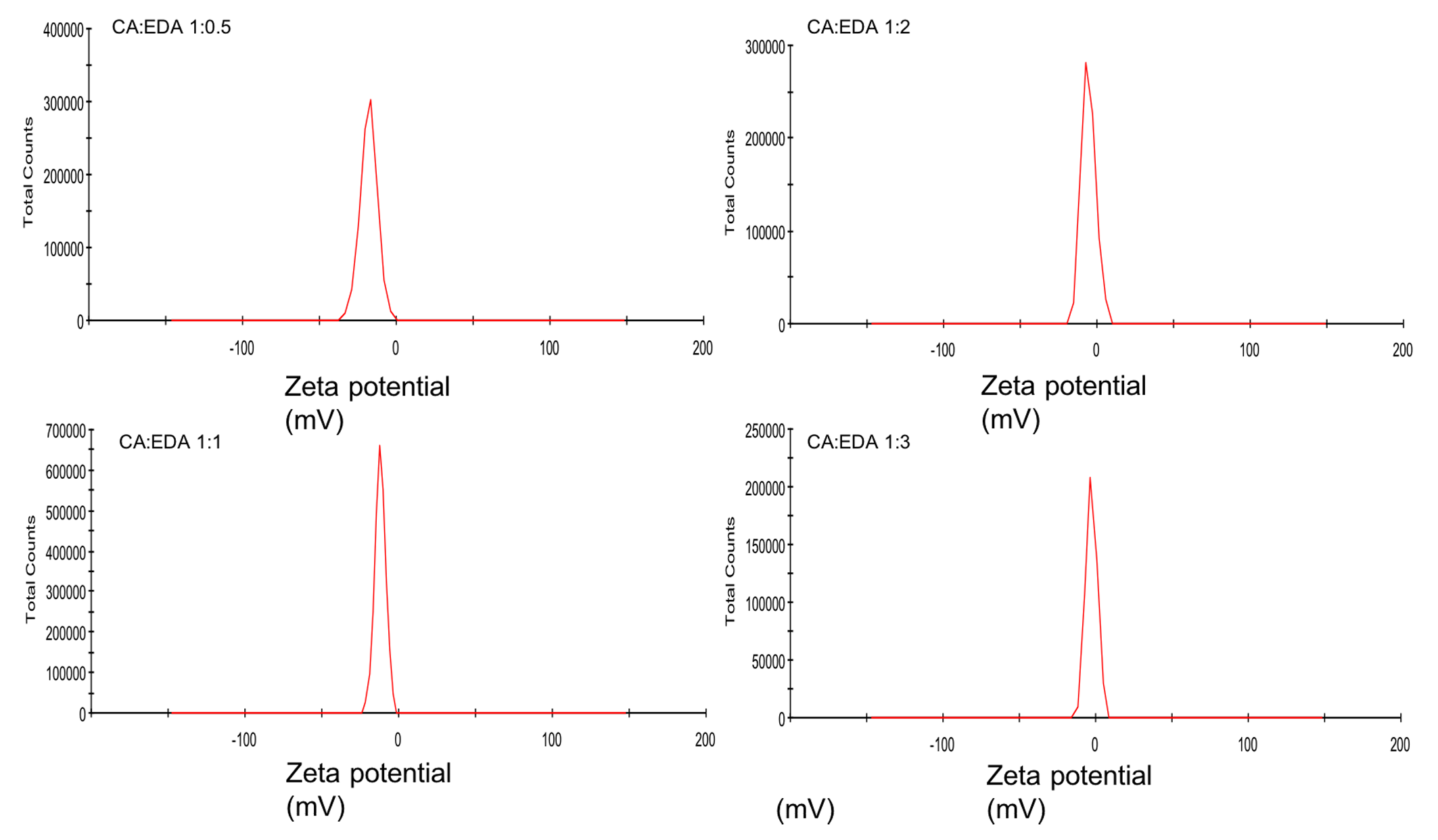

Supplement: S1 Fig — (TIF) [file pone.0216230.s001.tif]

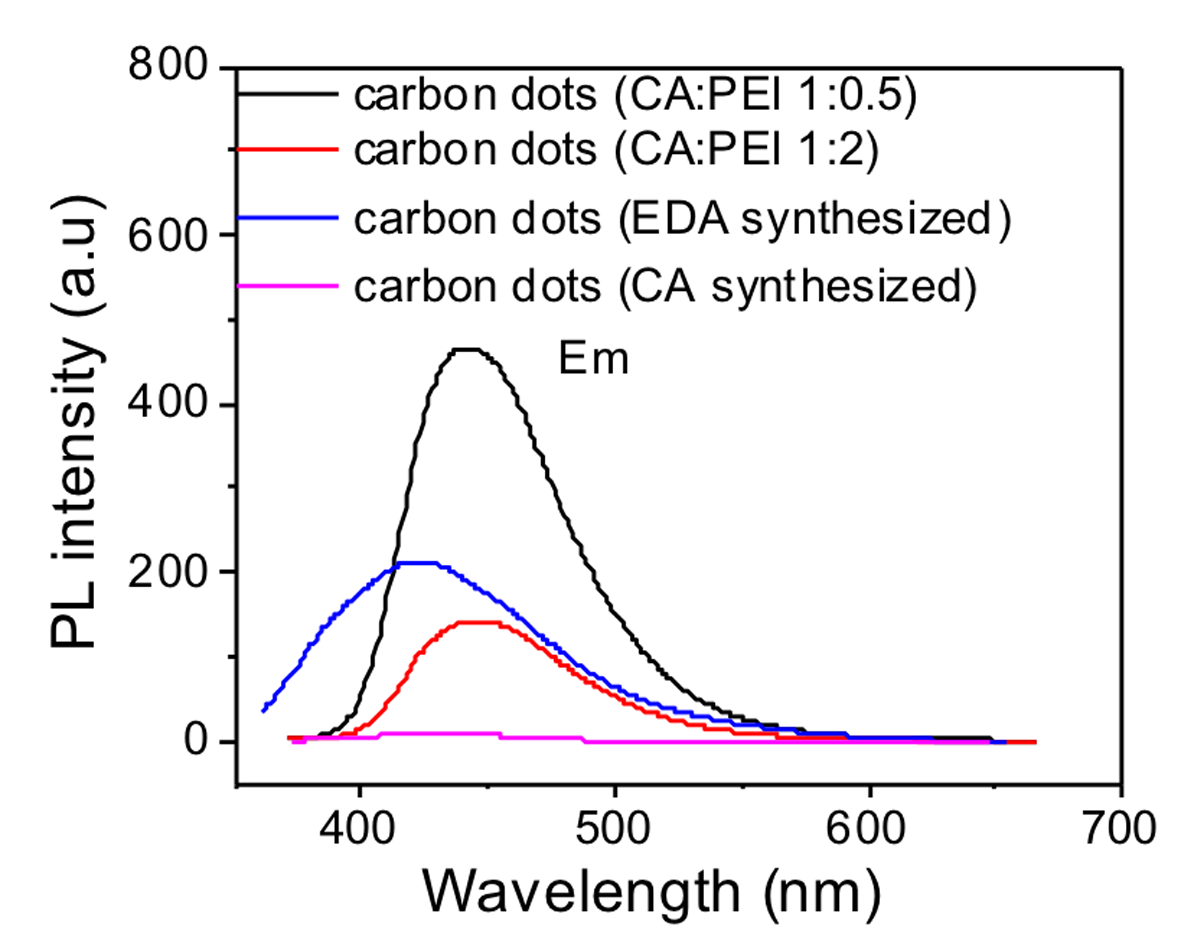

Supplement: S2 Fig — (TIF) [file pone.0216230.s002.tif]
